# Laboratory colonization stabilizes the naturally dynamic microbiome composition of field collected *Dermacentor andersoni* ticks

**DOI:** 10.1186/s40168-017-0352-9

**Published:** 2017-10-04

**Authors:** Cory A. Gall, Glen A. Scoles, Krisztian Magori, Kathleen L. Mason, Kelly A. Brayton

**Affiliations:** 1 Department of Veterinary Microbiology and Pathology, Washington State University, Pullman, WA 99164-7040 USA; 2 Paul G. Allen School for Global Animal Health, Washington State University, Pullman, WA 99164-7040 USA; 3 Animal Disease Research Unit, US Department of Agriculture, Agricultural Research Service, Washington State University, Pullman, WA 99164-6630 USA; 4 Department of Biology, Eastern Washington University, Cheney, WA 99004-2445 USA

**Keywords:** Endosymbiont, Ecology, Community, Symbiosis

## Abstract

**Background:**

Nearly a quarter of emerging infectious diseases identified in the last century are arthropod-borne. Although ticks and insects can carry pathogenic microorganisms, non-pathogenic microbes make up the majority of their microbial communities. The majority of tick microbiome research has had a focus on discovery and description; very few studies have analyzed the ecological context and functional responses of the bacterial microbiome of ticks. The goal of this analysis was to characterize the stability of the bacterial microbiome of *Dermacentor andersoni* ticks between generations and two populations within a species.

**Methods:**

The bacterial microbiome of *D. andersoni* midguts and salivary glands was analyzed from populations collected at two different ecologically distinct sites by comparing field (F1) and lab-reared populations (F1-F3) over three generations. The microbiome composition of pooled and individual samples was analyzed by sequencing nearly full-length 16S rRNA gene amplicons using a Pacific Biosciences CCS platform that allows identification of bacteria to the species level.

**Findings:**

In this study, we found that the *D. andersoni* microbiome was distinct in different geographic populations and was tissue specific, differing between the midgut and the salivary gland, over multiple generations. Additionally, our study showed that the microbiomes of laboratory-reared populations were not necessarily representative of their respective field populations. Furthermore, we demonstrated that the microbiome of a few individual ticks does not represent the microbiome composition at the population level.

**Conclusions:**

We demonstrated that the bacterial microbiome of *D. andersoni* was complex over three generations and specific to tick tissue (midgut vs. salivary glands) as well as geographic location (Burns, Oregon vs. Lake Como, Montana vs. laboratory setting). These results provide evidence that habitat of the tick population is a vital component of the complexity of the bacterial microbiome of ticks, and that the microbiome of lab colonies may not allow for comparative analyses with field populations. A broader understanding of microbiome variation will be required if we are to employ manipulation of the microbiome as a method for interfering with acquisition and transmission of tick-borne pathogens.

**Electronic supplementary material:**

The online version of this article (10.1186/s40168-017-0352-9) contains supplementary material, which is available to authorized users.

## Background

Arthropods have the largest biodiversity in the animal kingdom, and although there are over 1 million described arthropod species, only a small proportion of them transmit pathogenic microorganisms [1]. Nevertheless, nearly a quarter of emerging infectious diseases identified in the last century were transmitted by arthropods [2]. Though arthropods can carry pathogenic microorganisms, they are colonized primarily by non-pathogenic microbes, termed endosymbionts, with pathogenic species making up only a small proportion, if any, of the composition [3–5]. The literature suggests that these endosymbionts may play essential roles in the physiology and fitness of the arthropod, by providing dietary supplementation or affecting things such as survival, fecundity, and innate immune responses against pathogens, which includes inhibition of acquisition and transmission [4, 6–8]. Several mechanisms for inhibiting pathogens using endosymbionts have been proposed; however, the effectiveness of these mechanisms differs based on several factors, such as the pathogen system, the endosymbiont being targeted, the ecology of the arthropod, and pathogen dynamics [9–14]. Nevertheless, in the last decade, manipulation of endosymbiont populations has been exploited in several systems to decrease vector fitness or pathogen transmission competence.

Though the microbiome has been increasingly studied with the advances in sequencing, the majority of arthropod microbiomes studied have been medically- or agriculturally relevant insects with a lack of in-depth study of non-insect arthropods [15–19]. Ticks, which are of medical-veterinary importance due to their vector capacity, are responsible for transmitting at least 14 known pathogens in the USA, with several new and emerging tick-borne pathogens recently identified [20–25]. To date, the bulk of tick microbiome research has focused on discovery and description, with few studies analyzing the ecology and fluctuations of the bacterial microbiome [14, 26–29].

The current literature has conflicting reports on the importance of the environment in shaping the tick microbiome [27, 30–32], though this may be due to limited knowledge of the ecology of the microbiome in ticks. Recently, our lab has demonstrated that the Rocky Mountain wood tick, *Dermacentor andersoni*, has differences between populations in microbial composition based on tissue type within the tick and geographic location of the population; however, we sampled ticks from the field once and the bulk of our data was collected from laboratory reared ticks [14, 33]. It has been well documented that biotic and abiotic environmental factors, such as host migration and climate change, have significant effects on arthropods and their associated pathogens [34–36]; however, most of these ecological studies have ignored the environmental influence on the non-pathogenic endosymbiont composition [37, 38]. There have been several articles proposing the importance of the microbiome with regard to tick-borne pathogens, but there has not been a sufficient amount of research into the ecological responses, spatial variation, or environmental influence of these microbial communities to truly understand the response of the microbial community [19]. The ecology of the tick microbiome needs to be more fully understood if this type of work is to lead to manipulation of the microbiome as a novel method for biocontrol of tick-borne pathogens.

To address this knowledge gap, we compared the microbiome of F1 *D. andersoni* ticks over multiple years from two ecologically different geographic locations to multiple generations of lab-reared ticks from the same populations to identify the relatability of the microbiome of lab to field tick populations. Currently, tick microbiome studies have focused on either laboratory-reared ticks or field-collected ticks; and without investigating the influence of lab rearing, there is a potential disconnect between comparisons of microbiome data from lab and field collected ticks. In addition, we were interested in investigating the “core” microbiome of *D. andersoni*, which consists of symbionts that are transovarially (mother to offspring) transmitted. To eliminate symbionts that were acquired through environmental exposure, we analyzed the microbiome of field-collected ticks that were reared in the lab for one generation (F1). Lastly, we compared the microbiome of individual ticks collected directly from the field with individual ticks reared one generation in the laboratory, as well as comparing individuals with that of pooled ticks to develop an understanding of variation of individual ticks within the population. We hypothesized that the microbiome of *D. andersoni* would stabilize when reared in a “simplistic” laboratory setting.

## Methods

### Tick collection and laboratory rearing

**Fig. 1:**
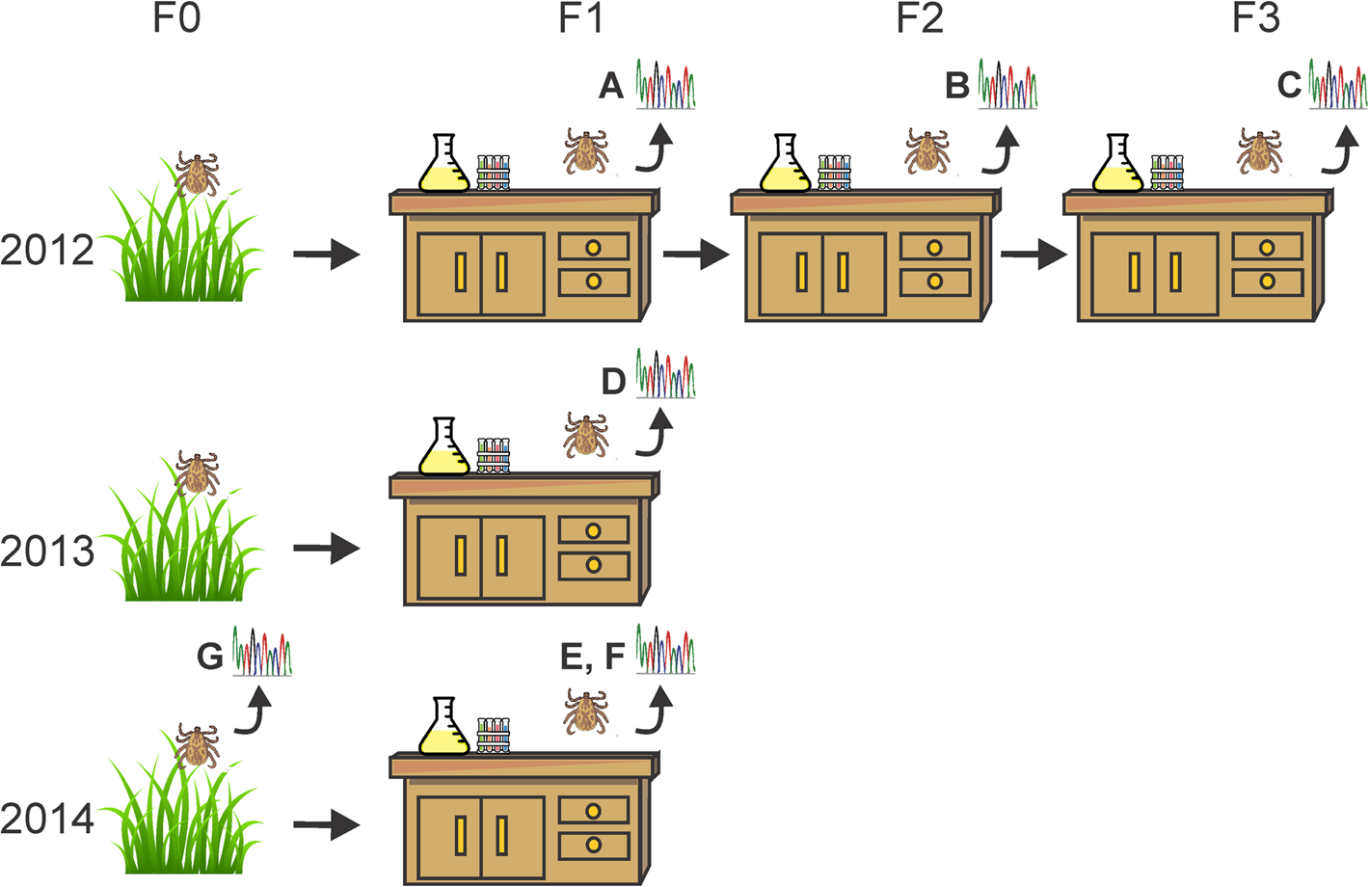
Experimental design for the comparison between field collected and lab-reared ticks. Ticks were collected from the field in Burns, OR and Lake Como, MT each spring during 2012–2014 (F0) and reared one generation (F1) in the lab. In this study we focused on the F1 generation as it is considered to most closely represent field collected ticks, with the exception that the microbiome contains only those endosymbionts that are transovarially passaged. The initial cohort that was collected in 2012 was used to establish a laboratory colony that was perpetuated for three generations. In 2013 and 2014, the field collected ticks were only reared to the F1 stage for microbiome sampling and comparison to the 2012 cohort; these lines were not continued. All ticks were dissected and MG and SG were assayed for bacterial microbiome resulting in seven (×2 for each tissue) sequence sets. **a**, **d**, and **e** correspond to the F1 microbiome from 2012, 2013 and 2014, respectively, and are considered as field collected microbiome data. **b** and **c** correspond to the F2 and F3 microbiome from the 2012 laboratory colonized ticks. Data for groups A-E was acquired by pooling tissues (MG or SG) from 30 ticks, and 3 biological replicates were performed—i.e., in all, 90 ticks were analyzed per group. In 2014, we analyzed individual ticks at the F0 and F1 stage resulting in data set **f** (F1) and **g** (F0). Each horizontal arrow indicates a generational rearing which takes approximately 1 year to complete

Questing adult *D. andersoni* were collected by dragging a 1-m^2^ cloth over vegetation at sites in Burns, Oregon and Lake Como, Montana each spring from 2012 to 2014 [14, 39]. *D. andersoni* ticks have a multi-year life cycle and collecting adult ticks as soon as adult ticks begin to emerge (immediately after spring snow clears) allows us to sample the adult population for that given year. Field collected ticks (a minimum of 100 males and 100 females) were used to establish laboratory colonies, which were maintained at the Holm Research Center, USDA, ARS Animal Disease Research Unit in Moscow, Idaho (Fig. 1). These colonies were reared using Sprague-Dawley rats for larval feedings and Holstein calves for nymphal and adult feedings, and in total required approximately 12 months to rear each generation, which is the shortest amount of time *D. andersoni* can complete one full life cycle in the laboratory, and shorter than in nature (Washington State University IACUC #04440–004 and University of Idaho IACUC #2013–66) [39]. The host bloodmeal has been shown to have no effect on the arthropod microbiome [30], unless there is an active pathogenic infection in the host. To ensure microorganisms were not introduced into the tick microbiome, we screened bovine blood used in our experiments for the presence of bacteria by running PCR with universal 16S rRNA gene primers. The laboratory setting allows for the tick microbiome to be analyzed in a “simple”, clean system, with the absence of harsh environmental conditions. The majority of this study focuses on microbiome analysis of F1 ticks, one generation removed from the field, which allows us to analyze microbiome components that are transovarially transferred and are therefore considered important for some aspect of the biology of the tick. For the purpose of this study, we are considering F1 ticks as field ticks.

For tick microbiome analysis, a cohort of adult male ticks (100 individuals) from each colony were fed on a Holstein calf for 7 days and dissected to collect midguts (MG) and salivary glands (SG) for genomic DNA isolation. The microbiome analysis focused on the MG and SG tissues due to their role in pathogen acquisition and transmission [40]. Adult male ticks were analyzed because they are the primary means of *Anaplasma marginale* transmission (a pathogen we study in our lab, but which is not the focus of this work), which occurs when male ticks move from infected to naïve hosts in search of mating opportunities [41].

### DNA preparation and sequencing

Adult male ticks were fed for 7 days and then dissected within 24 h. Before dissection, the ticks were surface sterilized by triple rinsing: shaking ticks in tick wash (0.25% EtOH, 0.25% bleach) for 1 min followed by a double 1 min rinse of ddH_2_0. All dissection tools were sterilized between each dissection [39]. Tick MG and SG were dissected and either pooled in groups of 30 with three biological replicates or collected as individuals (*n* = 5 for Burns F0 and F1 and *n* = 6 for Lake Como F0 and F1). Tissues were stored in Cell Lysis Solution (Qiagen, Valencia, CA) and Proteinase K (1.25 mg/ml). Genomic DNA was isolated using the PureGene Extraction kit (Qiagen) according to the manufacturer’s specifications. Platinum Pfx DNA Polymerase (Invitrogen, Carlsbad, CA) was used to amplify the 16S rRNA genes following the manufacture’s amplification protocol and the primers described below at an annealing temperature of 54 °C with the following thermocycler reaction:

$$ \left[\frac{94^{{}^{\circ}}C}{5^{\prime }}\right]\frac{\left[\frac{94^{{}^{\circ}}C\kern1.75em {54}^{{}^{\circ}}C\kern1.75em {68}^{{}^{\circ}}C\ }{15^{"}\kern2.5em {30}^{"}\kern2.25em {1.5}^{\prime}\kern0.5em }\right]}{5x}\frac{\left[\frac{94^{{}^{\circ}}C\kern2em {68}^{{}^{\circ}}C}{15^{"}\kern2em {1.5}^{\prime }}\right]}{30x}\left[\frac{68^{{}^{\circ}}C}{\infty}\right] $$

Each sample was amplified in at least three technical replicates with the same sample barcode. In order to pool our samples, we used barcoded sample-specific primers following the Amplicon Fusion Primer format [5]. To target as many 16S rRNA gene variable regions as technically possible, we used modified universal primers 27F (AGAGTTTGATCMTGGCTCAGAACG) and 1435R (CGATTACTAGCGATTCCRRCTTCA) [14]. Prior to sequencing, the DNA concentration of each sample was measured with a bioanalyzer (Agilent, Santa Clara, CA) and samples were pooled in equimolar amounts. Samples were submitted to Washington State University’s Sequencing Core for Pacific Biosciences (PacBio, Menlo Park, CA) Circular Consensus Sequencing (CCS), which provides a > 99.9999% accuracy.

### Sequence analysis

Raw sequence data were filtered by PacBio software according to the expected sequence size range and 99% accuracy, in order to filter out poor quality sequence reads. After PacBio internal filtering, reads were analyzed with Ribosomal Database Program (RDP; 42) to classify to genus level using 95% confidence interval (CI). Additionally, filtered reads were blasted against the NCBI BLASTn database to identity to species level taxonomic classification. Blast results were filtered at a minimum of 1275 bp in length and 98% identity. Reads that had 98% identity and above were classified at the species level [30, 42–44], and reads that had less than 98% identity were recorded at lower taxonomic classification. Operational taxonomic units (OTUs) that accounted for 1% or less of the total proportion of the microbiome were grouped together in a “rare” category. Sequence reads that did not meet 95% CI were grouped into an “unclassified” category.

The average number of reads/sample after filtering was 2857 sequences, which satisfied a rarefaction curve (see below) allowing us to be confident that all OTUs were captured. Raw microbiome sequence data were deposited in the sequence read archive at National Center for Biotechnology Information (Additional file 1).

### Statistical analysis

The microbiome compositions were analyzed using R (RStudio V. 0.99.486) with the open source *vegan* package (v. 2.4–0) and *phyloseq* microbiome package (v. 1.16.2), a statistical package specifically designed for reproducible analysis of complex microbiome data that has been used to analyze microbiome data from numerous organisms [45–50]. *Phyloseq* was used to import sample data, build a data matrix, and to calculate microbiome differences between and within samples (See Additional file 2 for R scripts). Furthermore, rarefaction curves were calculated using the *phyloseq* package with 1000 iterations to determine the minimum number of reads per sample necessary for optimal coverage. Statistical analysis was conducted using permutational MANOVA and pairwise comparisons with 999 permutations within the “adonis” function in *vegan*, with Bray-Curtis dissimilarity indices [51, 52], as well as Non-metric multidimensional distance analysis to identify microbiome differences between and within samples, using the “ordinate” function [26]. Q values, which take into account false discovery rate, and F-associated *p* values were used to determine statistical significance [51].

## Findings

### Sequence results

Microbiome samples were sequenced using a PacBio CCS SMRT platform. After raw reads were filtered and trimmed based on quality and length, there was an average of 2857 sequence reads/sample, which satisfied the rarefaction curve (several typical rarefaction curves are shown in Additional file 3) allowing us to be confident that all OTUs were captured. The average read length of 16S rRNA gene sequences was 1310 base pairs. We found a mean of nine major OTUs (excluding OTUs grouped into “rare”) in a given microbiome analysis. Our lab has demonstrated that *D. andersoni* has a low OTU count using both Roche 454 and PacBio CCS sequencing [14, 32].

### Analysis of the bacterial microbiome of F1 field populations

**Fig. 2:**
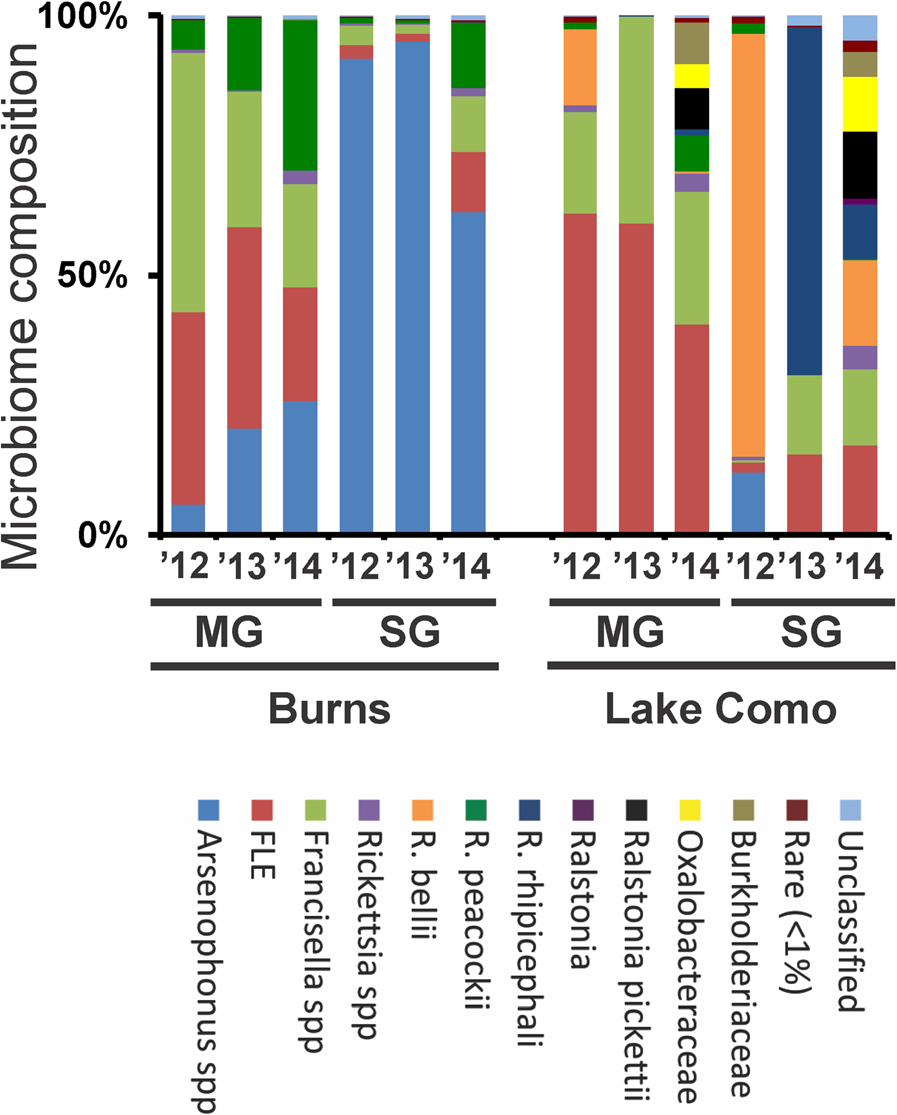
Microbiome of *D. andersoni* ticks in field-caught populations. The bacterial composition of the midgut (MG) and salivary glands (SG) of adult male Burns (B) and Lake Como (LC) ticks was characterized in F1 ticks from 2012 to 2014. Taxa detected included *Arsenophonus spp.* (blue), *FLE* (red), *Francisella spp.* (light green), *Rickettsia spp.* (purple), *R. bellii* (orange), *R. peacockii* (dark green), *R. rhipicephali* (dark blue), *Ralstonia spp.* (dark purple), *Ralstonia pickettii* (black), Oxalobacteraceae (yellow), Burkholderiaceae (light brown), rare (dark red), and unclassified (light blue)

We collected ticks from two field sites for three consecutive years. After field collection, ticks were reared in the lab to produce F1 populations, which allowed us to have sufficient ticks to work with. The bacterial microbiome of both Burns and Lake Como F1 field populations were complex and differed based on tissue type and geographic location each year of this study. The endosymbionts that differed significantly between locations over the sampled years included: *Arsenophonus spp.* (*q < 0.001), Rickettsia bellii* (*q < 0.005*)*,* and *R. rhipicephali* (*q < 0.010*; Fig. 2)*.* Furthermore, the composition of the microbiome differed between the MG and SG for both populations over the sampling period (*F*_*(*2)_ = 2.55, *p = 0.014;* Fig. 2).

*The core microbiome of Burns ticks remained the same over the course of the study:* The proportions of the minor endosymbionts in the Burns population shifted during 2012–2014, however the core microbiome was conserved, which included: *Arsenophonus spp.* in the SG*,* and *Arsenophonus spp., Francisella*-like endosymbiont (FLE), *Francisella spp*., and *R. peacockii* in the MG (Fig. 2). The microbiome of Burns did not significantly change in the MG between 2012 and 2014 (*F*_*(*2)_ = 1.81, *p = 0.101*); however, the SG experienced significant changes (*F*_*(*2)_ = 4.65, *p = 0.012*).

*The microbiome of Lake Como ticks differed each year:* The microbiome of Lake Como ticks was significantly different each sampling year in the SG (*F*_*(*2)_ = 5.16, *p = 0.003*), with minor, but significant, changes in the MG (*F*_*(*2)_ = 3.53, *p = 0.017;* Fig. 2). The major endosymbiont in the SG of Lake Como ticks was *R. bellii* in 2012, *R. rhipicephali* in 2013, and there was not a dominating endosymbiont in 2014.

### Analysis of the bacterial microbiome of lab-reared populations

**Fig. 3:**
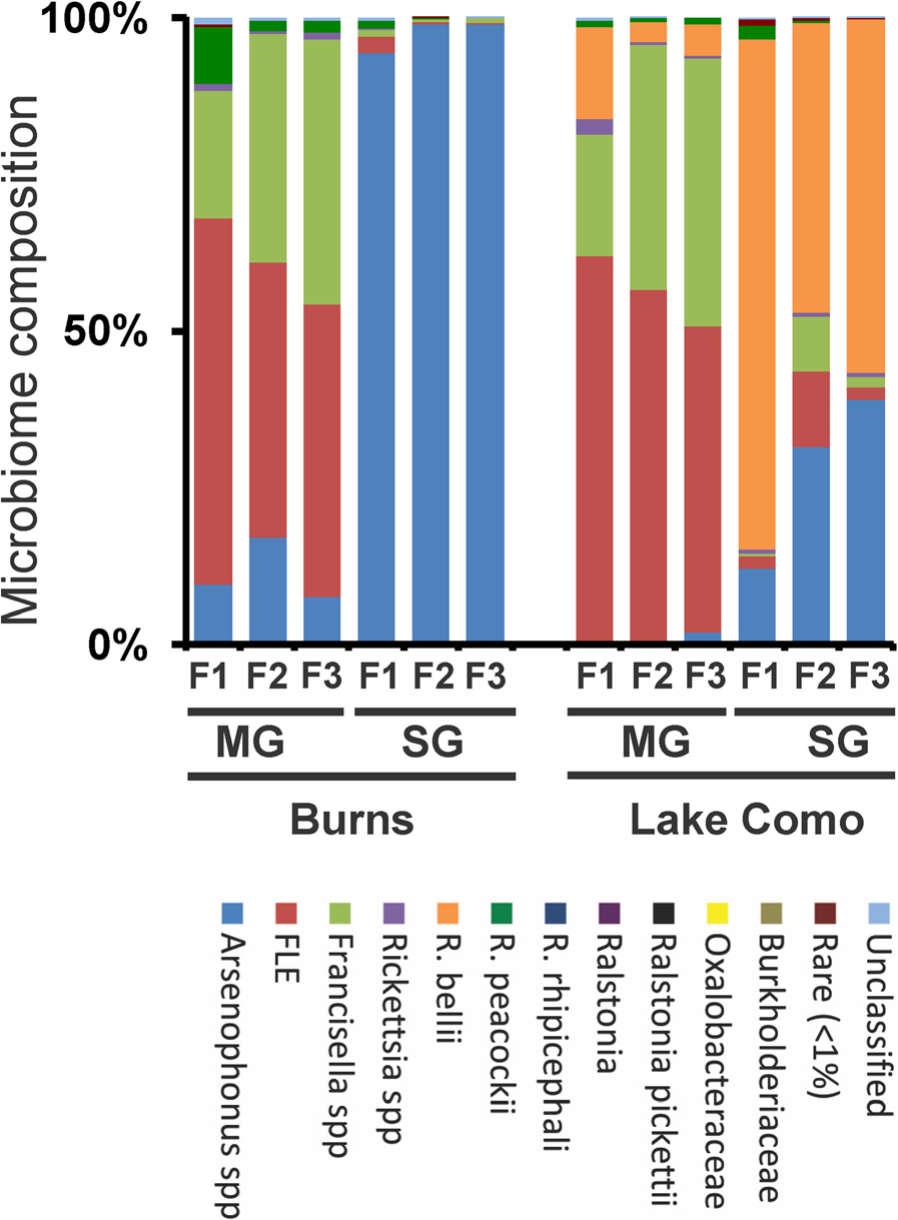
Microbiome of *D. andersoni* ticks in lab-reared populations. The bacterial composition of the midgut (MG) and salivary glands (SG) of adult male Burns (B) and Lake Como (LC) ticks was characterized in lab-reared ticks from F1–F3 generations. Species/genera detected included *Arsenophonus spp.* (blue), *FLE* (red), *Francisella spp.* (light green), *Rickettsia spp.* (purple), *R. bellii* (orange), *R. peacockii* (dark green), *R. rhipicephali* (dark blue), *Ralstonia spp.* (dark purple), *Ralstonia pickettii* (black), Oxalobacteraceae (yellow), Burkholderiaceae (light brown), rare (dark red), and unclassified (light blue)

The bacterial microbiome of Burns and Lake Como lab populations (F1-F3) were consistently different between tissue types over the three generations (*F*_*(*2)_ = 2.70, *p = 0.006* and *F*_*(*2)_ = 22.23, *p = 0.001*, respectively; Fig. 3). The endosymbionts that differed significantly between the Burns MG and SG over the generations included: *Arsenophonus spp.* (*q < 0.005), R. peacockii* (*q < 0.005*)*, Rickettsia spp.* (*q < 0.005*)*, Francisella spp.* (*q < 0.001*)*,* and FLE (*q < 0.001*)*.* The endosymbionts that differed between Lake Como MG and SG included: FLE (*q < 0.002*)*, Francisella spp.* (*q < 0.005*)*, R. bellii* (*q < 0.005*)*,* and rare (*q < 0.006;* Fig. 3). However, the interactions between generation and tissue type were not significant for the Burns or Lake Como populations (*F*_*(*2)_ = 1.75, *p = 0.088* and *F*_*(*2)_ = 1.64, *p = 0.178*, respectively), meaning that the differences in the bacterial microbiome between tissue types were consistent across generations.

### Bacterial analysis of individual ticks

**Fig. 4:**
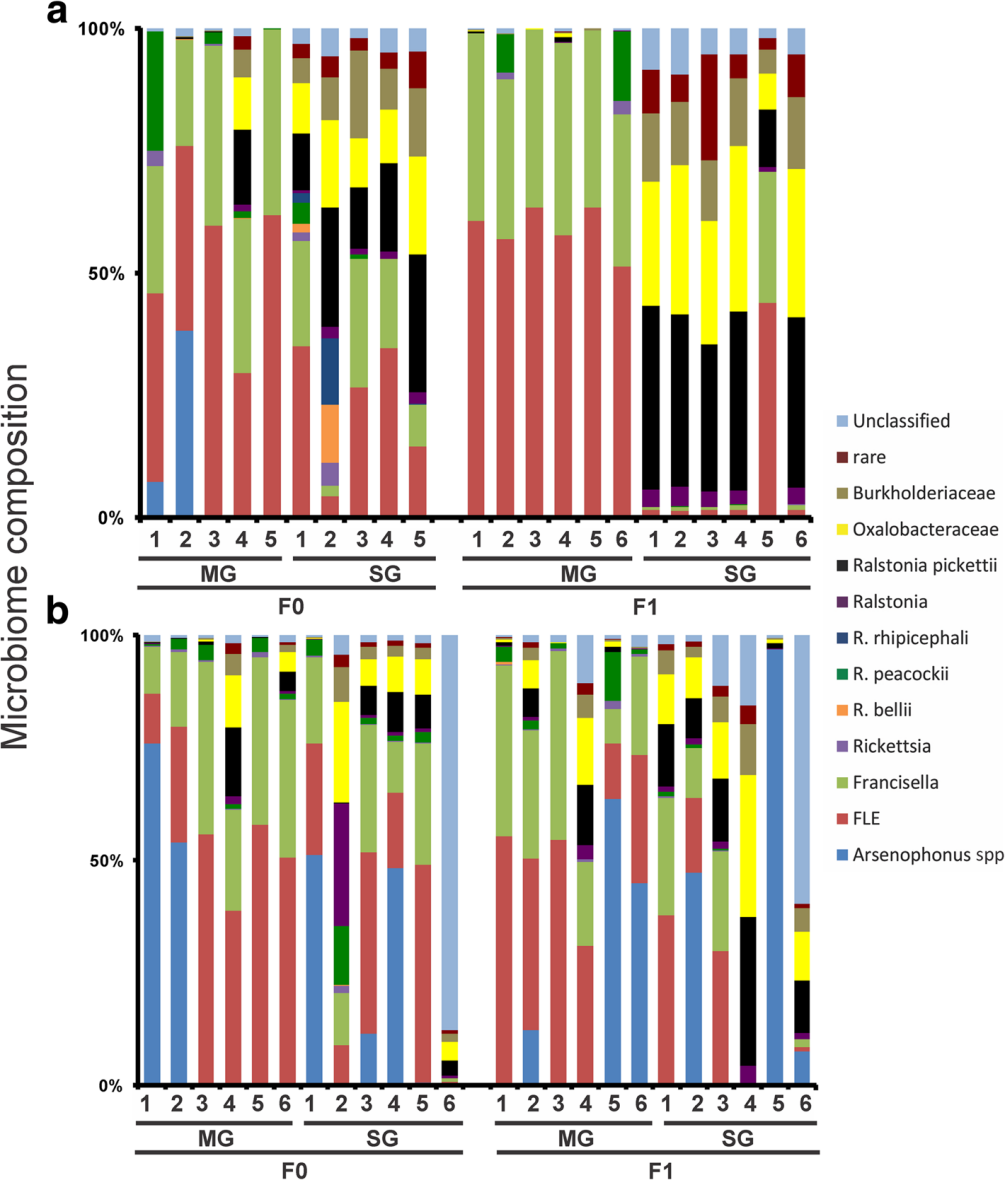
Microbiome of *D. andersoni* ticks in field-caught and lab-reared individuals. The bacterial composition of the midgut (MG) and salivary glands (SG) of adult male (**a**) Burns and (**b**) Lake Como ticks was characterized in F0 and F1 individual ticks from 2014. Species/genera detected included *Arsenophonus spp.* (blue), *FLE* (red), *Francisella spp.* (light green), *Rickettsia spp.* (purple), *R. bellii* (orange), *R. peacockii* (dark green), *R. rhipicephali* (dark blue), *Ralstonia spp.* (dark purple), *Ralstonia pickettii* (black), Oxalobacteraceae (yellow), Burkholderiaceae (light brown), rare (dark red), and unclassified (light blue)

A preliminary analysis of the bacterial microbiome of five to six individual Burns and Lake Como F0 and F1 ticks was conducted to analyze microbiome changes in response to laboratory rearing. When excluding generation, Burns and Lake Como ticks had significant differences in the bacterial composition between the MG and SG (*F*_(1)_ = 2.92, *p = 0.023* and *F*_(1)_ = 33.57, *p = 0.001*, respectively; Fig. 4). However, there were not any significant differences in individual endosymbionts between F0 and F1 in the Burns or Lake Como ticks. Furthermore, there were not significant interactions between generation and tissue type in either Burns or Lake Como individual ticks (*F*_*(*1)_ = 0.38, *p = 0.843* and *F*_*(*1)_ = 3.08, *p = 0.064*, respectively). Thus, the differences between tissue types are consistent between generations, including the field to lab transition. Although robust statistical analyses cannot be performed due to differences in sample sizes, it appears that the microbiome composition of six individuals does not reflect the composition of the microbiome of three pools of tissues from 30 ticks.

## Discussion

The microbiomes of several medically relevant species of ticks have previously been characterized. Further development of the concept of using manipulation of the microbiome as a novel means for pathogen and/or vector control requires that the focus of research efforts move past general characterization and towards understanding the microbiome as an ecological system that responds to changing conditions. We have little understanding of the role of the tick microbiome, however evidence that it “behaves” differently under different conditions suggests a functional response. A few studies have begun to investigate the ecology of the tick microbiome [26, 30, 32]; however, there are still many unknown factors and this area of research remains a “black box”. The focus of this study was to address a gap in the understanding of the stability of the microbiome of *D. andersoni* over three generations in two ecologically diverse populations. The data presented in this study provides evidence that tick habitat (Burns vs. Lake Como vs. the laboratory setting) has an influence on the composition and stability of the microbiome; thus, we accept our hypothesis that the microbiome of *D. andersoni* will stabilize when reared in the laboratory setting.

We are interested in population scale microbiome analysis, as we believe that to effect change we will need to work at the population level rather than at the level of the individual. Ergo, we are interested in the metapopulation that dominates within a given tick population. Our analysis differs from other published tick analyses: we typically find many fewer OTUs than do other researchers when examining ticks. We feel this is due to two factors: (1) we employ long sequence read technology with very high accuracy that allows better discrimination of the 16S rRNA gene, and (2) we are examining dissected tissues rather than whole ticks, which may eliminate some elements of the total (whole) tick microbiome. An alternative explanation could be that this tick has a more simplistic microbiome than other ticks. Although other researchers have not examined this tick, and therefore we cannot compare our data to that acquired by other researchers, we have used two different technologies to obtain our sequence data and both have yielded low OTU compositions for this tick species.

Identifying habitat influences on the tick microbiome has not been trivial nor have the results of previous studies been consistent; however, species-specific differences in microbiomes are evident when comparing the literature [14, 26, 32, 52, 53]. Studies have shown that transmission of tick-borne pathogens can differ based on geographic location of the vector [39, 54]; yet, intraspecies microbiome variation has been less well studied. Although there is evidence that geographic-specific intraspecies variation in the composition of the microbiome occurs [14, 32, 53, 54], many of these studies have combined tick collection years, pooled life stages, or have had low sample sizes, which has led to equivocal conclusions. Our results showed that the microbiome of *D. andersoni* differed based on geographic location of the tick population; the microbiome was consistently different between ticks from the Burns and Lake Como sites, irrespective of generation or source of the population (field or lab; Figs. 2 and 3).

Additionally, the geographic differences described here included tissue-specific differences in compositions as seen in the MG and SG of *D. andersoni*. The tissue-specific differences in microbiomes were consistent over each of the three consecutive field seasons as well as the three lab generations in both Burns and Lake Como populations (Figs. 2 and 3). These findings support previous microbiome studies in several species of ticks [4, 44], indicating a dynamic host-microbiome relationship that is dependent on the in vivo habitat. It is unknown whether the reason for differences in the composition between different tissues is physical space, nutrient availability, immune evasion, or some other mechanism. Irrespective of the reason for microbiome differences between tissue types, this suggests that there could be three different approaches for using manipulation of the microbiome to target pathogens: non-specific, which would target all tissues; MG-specific manipulation targeting pathogen acquisition; or SG-specific targeting pathogen transmission.

However, intraspecies variation of the tick microbiome at the individual level as well as at the population level needs to be thoroughly investigated before manipulation of the microbiome can be considered as a possible biocontrol method. Microbiome variation within tick populations has been given little focus. To fully understand the dynamics of the microbial community, variations between microbial compositions on different scales needs further investigation. To date, sample sizes in previous tick microbiome studies have been inconsistent, ranging in some studies from a few individuals to pools of many ticks in others. In order to appropriately characterize the microbiomes of species and populations of ticks, the degree of variation needs to be thoroughly understood. Variation between individual ticks has been shown to occur in several species [4, 26, 54]; however, there has not been a comparison between the compositions of the microbiome of individual ticks and pools of ticks (population-level) to find an ideal number of individuals that needs to be sampled to accurately represent the population as a whole. We characterized the microbiome of pooled ticks (population level) as well as completed a preliminary analysis of individual ticks; however, as the individual tick results appear to show, the microbiome composition of six individuals does not represent the microbiome of the population (Fig. 4). Although there was not a significant difference between individuals, the most noticeable difference was that the rare OTUs become less distinct in the microbiome of the whole population as compared to the individual ticks. However, to effectively exploit manipulation of the microbiome for biocontrol, it will be important to target endosymbionts that are conserved at the population level; these will likely be endosymbionts that are essential for tick fitness and physiology. Interestingly, we note that individual ticks with microbiome compositions that differ from the population as a whole may represent a source of epigenetic heterogeneity on which selection can act to adapt the population to changing biotic or abiotic factors.

The influence and long-term effect of lab colonization on the tick microbiome is a relative unknown. The effect of lab rearing on the microbiome composition deserves thorough investigation in order to be able to understand how results from lab studies apply to field populations. The majority of tick microbiome studies have used either field-caught or lab-reared ticks; however, without knowing the effect of lab colonization on the bacterial community, it is difficult to directly compare microbiome compositions from the two sources. The microbiome of *Amblyomma maculatum* has previously been shown to be different between lab colonies and field-caught ticks [44], though this study combined ticks from multiple locations and over two field collection years. Although Budachetri and colleagues (2014) found significant microbiome differences between the tick sources, the results from their study do not reveal the effect of environmental factors, such as possible microbiome variation due to location and/or year of sampling. Our results (Figs. 2 and 3) showed that the microbiome of both Burns and Lake Como lab populations retain the same composition for three generations as the original ticks that were used as founders of the lab colony. The consistency of the microbiome of the Burns population is ideal for conducting lab-based microbiome experiments and will increase the reproducibility of these experiments; however, the ability to make comparisons between lab-reared and field caught ticks will depend on knowledge of the microbiome of wild-type ticks at the geographic location of the source of the tick population, as the results showed there were highly significant differences in the Lake Como populations when comparing lab and field tick microbiomes.

Understanding how the microbiome changes over time is one of the most important reasons for studying the ecology of the microbiome. Much of the current knowledge of the tick microbiome stems from compositional analyses made at a single point in time or from pooled samples collected in multiple years. Although understanding the microbiome at a given time is important, identifying microbiome differences within a species or population allows for the translation and adaptation of sequencing results into possible microbiome-targeting biocontrol strategies. For example, when comparing *D. andersoni* ticks from the Burns and Lake Como populations, location was important, as the microbiome of Burns ticks was consistent over time whereas the microbiome of Lake Como ticks varied significantly from year to year. A conserved microbiome is ideal for manipulation strategies, as an endosymbiont target would be consistent and endosymbiont-endosymbiont competition would be known; however, conservation of microbiome composition may be rare among tick populations [55]. Further investigation into a larger number of populations of *D. andersoni* is needed to fully understand the association between habitat conditions and host availability and microbiome changes.

## Conclusions

The ecology of the *D. andersoni* microbiome was dynamic and diverse. Though the degree of ecological variation was dependent on geographic location of the population, the microbiome of laboratory colonies may not allow for comparative analyses with field populations: laboratory colonization has a stabilizing influence on the microbiome, while the microbiome of ticks in the field continues to be subject to ecological influences. Furthermore, we have begun to explore population-specific microbiomes and how the microbiomes fluctuate with varying ecological exposure. Our major findings include:

1. The microbiome of laboratory-reared *D. andersoni* does not necessarily equate to the microbiome of *D. andersoni* collected from the field.
2. The bacterial microbiome of *D. andersoni* stabilized when reared in the laboratory setting.
3. The microbiome of Burns and Lake Como populations illustrated that the bacterial microbiome of different *D. andersoni* populations change differently over multiple generations.
4. The findings of our preliminary study comparing the microbiome of individual ticks collected directly from the field (F0) to individual ticks reared one generation in the lab (F1) demonstrated that there was not a difference between ticks removed from the field one generation.

While more populations of *D. andersoni* need to be analyzed, these results provide preliminary data for population-specific tick microbiomes, which may be used in the future as a source of population identification through microbiome “fingerprints.” As seen with unique microbiome fingerprints in humans [56], microbiome variation may be exploited in tick-borne disease epidemiology and modeling. Lastly, these results provide a template for experimental design for future tick microbiome studies, especially with regard to species level identification of the bacterial components of the microbiome.

## Additional files

Additional file 1: Accession numbers for sequence data. The raw sequence reads from the microbiome samples from this study were deposited in the National Center for Biotechnology Information database as SRR files. (DOCX 13 kb)

Additional file 2: R scripts for microbiome analysis. R was used to import sample data, build a data matrix, and the *phyloseq* and *vegan* packages were used to calculate microbiome differences between and within samples as well as rarefaction curves of the samples. (DOCX 34 kb)

Additional file 3: Representative rarefaction curves of *D. andersoni* ticks in 2012–2014 Lake Como populations. The species diversity of the midgut (MG) and salivary glands (SG) of adult male F1 Lake Como (LC) ticks collected from 2012 to 2014 were plotted as a function of read depth. Samples that were plotted include: 2014 SG (yellow), 2014 MG (dark blue), 2012 MG (red), 2012 SG (pale blue), 2013 SG (fuchsia) and 2013 MG (green). (DOCX 53 kb)

## Abbreviations

B: Burns, Oregon
FLE: *Francisella*-like endosymbiont
LC: Lake Como, Montana
MG: Midgut
OTU: Operational taxonomic unit
PacBio: Pacific Biosciences
RDP: Ribosomal Database Project
SG: Salivary glands
