# Supplementary material for: Laboratory colonization stabilizes the naturally dynamic microbiome composition of field collected Dermacentor andersoni ticks
Source: Microbiome. 2017 Oct 4;5:133. doi: 10.1186/s40168-017-0352-9 (PMC5628422; doi:10.1186/s40168-017-0352-9)
Supplement: Supplementary file 1 — Accession numbers for sequence data. The raw sequence reads from the microbiome samples from this study were deposited in the National Center for Biotechnology Information database as SRR files. (DOCX 13 kb) [file 40168_2017_352_MOESM1_ESM.docx]

**Additional File 1:** **Accession numbers for sequence data.** The raw sequence reads from the microbiome samples from this study were deposited in the National Center for Biotechnology Information database as SRR files.

STUDY: PRJNA317030 (SRP072719)

2012 F1 LC MG1 (SRR3325195)

2012 F1 LC MG2 (SRR3325232)

2012 F1 LC MG3 (SRR3325267)

2012 F1 LC SG1 (SRR3325338)

2012 F1 LC SG2 (SRR3325342)

2012 F1 LC SG3 (SRR3325343)

2012 F1 B MG1 (SRR3325344)

2012 F1 B MG2 (SRR3325345)

2012 F1 B MG3 (SRR3325350)

2012 F1 B SG1 (SRR3325347)

2012 F1 B SG2 (SRR3325348)

2012 F1 B SG3 (SRR3325349)

2012 F2 LC MG1 (SRR3325351)

2012 F2 LC MG2 (SRR3325352)

2012 F2 LC MG3 (SRR3325353)

2012 F2 LC SG1 (SRR3325354)

2012 F2 LC SG2 (SRR3325355)

2012 F2 LC SG3 (SRR3325356)

2012 F2 B MG1 (SRR3325357)

2012 F2 B MG2 (SRR3325358)

2012 F2 B MG3 (SRR3325346)

2012 F2 B SG1 (SRR3325359)

2012 F2 B SG2 (SRR3325470)

2012 F2 B SG3 (SRR3325482)

2012 F3 LC MG1 (SRR3325490)

2012 F3 LC MG2 (SRR3325491)

2012 F3 LC MG3 (SRR3325492)

2012 F3 LC SG1 (SRR3325498)

2012 F3 LC SG2 (SRR3325523)

2012 F3 LC SG3 (SRR3325525)

2012 F3 B MG1 (SRR3325527)

2012 F3 B MG2 (SRR3325528)

2012 F3 B MG3 (SRR3325529)

2012 F3 B SG1 (SRR3325530)

2012 F3 B SG2 (SRR3325531)

2012 F3 B SG3 (SRR3325532)

2013 F1 LC MG1 (SRR3325533)

2013 F1 LC MG2 (SRR3325534)

2013 F1 LC MG3 (SRR3325535)

2013 F1 LC SG1 (SRR3325536)

2013 F1 LC SG2 (SRR3325537)

2013 F1 LC SG3 (SRR3325538)

2013 F1 B MG1 (SRR3325539)

2013 F1 B MG2 (SRR3325540)

2013 F1 B MG3 (SRR3325541)

2013 F1 B SG1 (SRR3325542)

2013 F1 B SG2 (SRR3325543)

2013 F1 B SG3 (SRR3325544)

2014 F1 LC MG1 (SRR3325545)

2014 F1 LC MG2 (SRR3325546)

2014 F1 LC MG3 (SRR3325596)

2014 F1 LC SG1 (SRR3325598)

2014 F1 LC SG2 (SRR3325599)

2014 F1 LC SG3 (SRR3325602)

2014 F1 B MG1 (SRR3325604)

2014 F1 B MG2 (SRR3325627)

2014 F1 B MG3 (SRR3325628)

2014 F1 B SG1 (SRR3325629)

2014 F1 B SG2 (SRR3325630)

2014 F1 B SG3 (SRR3325631)
