# Supplementary material for: Laboratory colonization stabilizes the naturally dynamic microbiome composition of field collected Dermacentor andersoni ticks
Source: Microbiome. 2017 Oct 4;5:133. doi: 10.1186/s40168-017-0352-9 (PMC5628422; doi:10.1186/s40168-017-0352-9)
Supplement: Supplementary file 2 — R scripts for microbiome analysis. R was used to import sample data, build a data matrix, and the phyloseq and vegan packages were used to calculate microbiome differences between and within samples as well as rarefaction curves of the samples. (DOCX 34 kb) [file 40168_2017_352_MOESM2_ESM.docx]

**Additional File 2:** **R scripts for microbiome analysis.** R was used to import sample data, build a data matrix, and the *phyloseq* and *vegan* packages were used to calculate microbiome differences between and within samples as well as rarefaction curves of the samples.

**R scripts**

**Building data table**

>OTUmat=read.csv("F1_2012_2014_modified.csv",header=T)

>row.names(OTUmat)=OTUmat[,1]

>OTUmat=OTUmat[,-1]

>taxmat=read.csv("OTU_map.csv",header=T)

>taxmat=as.matrix(taxmat)

>rownames(taxmat)=rownames(OTUmat)

>OTU=otu_table(OTUmat,taxa_are_rows=TRUE)

>tax=tax_table(taxmat)

>Site=c(rep("Lake Como",6),rep("Burns",6),rep("Lake Como",6),rep("Burns",6),rep("Lake Como",6),rep("Burns",6))

>Year=c(rep("2012",12),rep("2013",12),rep("2014",12))

>Tissue=c(rep("SG",3),rep("MG",3),rep("SG",3),rep("MG",3),rep("SG",3),rep("MG",3),rep("SG",3),rep("MG",3),rep("SG",3),rep("MG",3),rep("SG",3),rep("MG",3))

>mysample_mat=matrix(cbind(Site,Year,Tissue),nrow=ncol(OTUmat),ncol=3)

>rownames(mysample_mat)=colnames(OTUmat)

>colnames(mysample_mat)=c("Site","Year","Tissue")

>mysample_mat=as.data.frame(mysample_mat)

>mysample_mat$Combined=paste(mysample_mat$Site,mysample_mat$Year,mysample_mat$Tissue)

>mysample=sample_data(as.data.frame(mysample_mat))

**Adonis analysis of data table**

>physeq=phyloseq(OTU,tax,mysample)

>plot_bar(physeq,fill="Species") + coord_flip()

>physeq2df=psmelt(physeq2)

>physeq2=transform_sample_counts(physeq,function(x) x/sum(x))

>plot_bar(physeq2,"Tissue",fill="Species") + facet_wrap(~Site*Year) + theme_bw() + scale_fill_discrete(breaks=c("Arsenophonus spp","FLE","Francisella spp", "Rickettsia spp", "R. bellii", "R. peacockii", "R. rhipicephali", "Ralsontia spp", "Ralstonia pickettii", "Oxalobacteraceae", "Burkholderiaceae", "Rare (<1%)", "Unclassified")) + theme(axis.title.y = element_blank()) + theme(axis.title.x = element_blank())

>p=plot_bar(physeq2,"Tissue",fill="Species") + facet_wrap(~Site*Year) + scale_fill_brewer(palette="Set3")

>p+geom_bar(aes(fill=Species,order=Species),stat="identity",position="stack")

**Ordinate analysis of data table**

>my_ord=ordinate(physeq,"NMDS")

>p1=plot_ordination(physeq,my_ord,shape="Tissue",color="Year") + geom_point(aes(size=Site)) +scale_size_manual(values=c(3,6))

>p1 + theme(panel.grid.major = element_blank(), panel.grid.minor = element_blank(), panel.background = element_blank(), axis.line = element_line(colour = "black"))

>print(p1)

>scores(my_ord)

>my_ord=ordinate(physeq,"NMDS")

>p1=plot_ordination(physeq,my_ord,shape="Tissue",color="Year") + geom_point(aes(size=Site)) +scale_size_manual(values=c(3,6))

>p1 + facet_wrap(~Tissue, 3)

>print(p1)

>scores(my_ord)

**Adonis to test differences**

>library(vegan)

>my_adonis=adonis(t(OTUmat) ~ Site*Year*Tissue,data=mysample_mat)

>my_adonis

**Pairwise differences LC SG 2012 vs LC MG 2012**

>my_adonis=adonis(t(OTUmat[,1:6]) ~ Tissue,data=mysample_mat[1:6,])

>my_adonis

**Pairwise differences LC SG 2012 vs LC SG 2013 vs LC SG 2014**

>my_adonis=adonis(t(OTUmat[,c(1:3,13:15,25:27)]) ~ Year,data=mysample_mat[c(1:3,13:15,25:27),])

>my_adonis

**Pairwise differences LC MG 2012 vs LC MG 2013 vs LC MG 2014**

>my_adonis=adonis(t(OTUmat[,c(4:6,16:18,28:30)]) ~ Year,data=mysample_mat[c(4:6,16:18,28:30),])

>my_adonis

**Pairwise differences whole tick LC 2012 vs LC 2013 vs LC 2014**

>my_adonis=adonis(t(OTUmat[,c(1:6,13:18,25:30)]) ~ Year,data=mysample_mat[c(1:6,13:18,25:30),])

>my_adonis

**Pairwise differences B SG 2012 vs B SG 2013 vs B SG 2014**

>my_adonis=adonis(t(OTUmat[,c(7:9,19:21,31:33)]) ~ Year,data=mysample_mat[c(7:9,19:21,31:33),])

>my_adonis

**Pairwise differences B MG 2012 vs B MG 2013 vs B MG 2014**

>my_adonis=adonis(t(OTUmat[,c(10:12,22:24,34:36)]) ~ Year,data=mysample_mat[c(10:12,22:24,34:36),])

>my_adonis

**Pairwise differences whole tick B 2012 vs B 2013 vs B 2014**

>my_adonis=adonis(t(OTUmat[,c(7:12,19:24,31:36)]) ~ Year,data=mysample_mat[c(7:12,19:24,31:36),])

>my_adonis

**Pairwise differences whole tick B 2012 vs LC 2012**

>my_adonis=adonis(t(OTUmat[,c(1:6, 7:12)]) ~ Site,data=mysample_mat[c(1:6, 7:12),])

>my_adonis

**Pairwise differences whole tick B 2013 vs LC 2013**

>my_adonis=adonis(t(OTUmat[,c(13:18, 19:24)]) ~ Site,data=mysample_mat[c(13:18, 19:24),])

>my_adonis

**Pairwise differences whole tick B 2014 vs LC 2014**

>my_adonis=adonis(t(OTUmat[,c(25:30, 31:36)]) ~ Site,data=mysample_mat[c(25:30, 31:36),])

>my_adonis

**Pairwise differences LC SG 2012 vs B SG 2012**

>my_adonis=adonis(t(OTUmat[,c(1:3,7:9)]) ~ Site,data=mysample_mat[c(1:3,7:9),],nperm=200)

>my_adonis

**Wilcoxon**

>multiple_tests=mt(physeq,"Site",test="wilcoxon")

>subset(multiple_tests,adjp<0.05)

**Lake Como F1-F3**

>OTUmat=read.csv("Lake_Como_F1_F3_modified.csv",header=T)

>row.names(OTUmat)=OTUmat[,1]

>OTUmat=OTUmat[,-1]

>taxmat=read.csv("OTU_map.csv",header=T)

>taxmat=as.matrix(taxmat)

>rownames(taxmat)=rownames(OTUmat)

>OTU=otu_table(OTUmat,taxa_are_rows=TRUE)

>tax=tax_table(taxmat)

>Site=c(rep("Lake Como",18))

>Year=c(rep("F1",6),rep("F2",6),rep("F3",6))

>Tissue=c(rep("SG",3),rep("MG",3),rep("SG",3),rep("MG",3),rep("SG",3),rep("MG",3))

>mysample_mat=matrix(cbind(Site,Year,Tissue),nrow=ncol(OTUmat),ncol=3)

>rownames(mysample_mat)=colnames(OTUmat)

>colnames(mysample_mat)=c("Site","Year","Tissue")

>mysample_mat=as.data.frame(mysample_mat)

>mysample=sample_data(as.data.frame(mysample_mat))

>physeq=phyloseq(OTU,tax,mysample)

>plot_bar(physeq,fill="Species")

>my_ord=ordinate(physeq,"NMDS")

>p1=plot_ordination(physeq,my_ord,shape="Year",color="Tissue") + geom_point(size=6) + scale_colour_manual(values=c("black","red"))

>print(p1)

>scores(my_ord)

**ADONIS on LC F1-F3**

>my_adonis=adonis(t(OTUmat) ~ Year*Tissue,data=mysample_mat)

>my_adonis

**LC F1-F3 within MG**

>my_adonis=adonis(t(OTUmat[,c(4:6,10:12,16:18)]) ~ Year,data=mysample_mat[c(4:6,10:12,16:18),])

>my_adonis

**LC within MG F1 vs F2**

>my_adonis=adonis(t(OTUmat[,c(4:6,10:12)]) ~ Year,data=mysample_mat[c(4:6,10:12),],perm=999)

>my_adonis

**LC F1-F3 within SG**

>my_adonis=adonis(t(OTUmat[,c(1:3,7:9,13:15)]) ~ Year,data=mysample_mat[c(1:3,7:9,13:15),])

>my_adonis

**Burns F1-F3**

>OTUmat=read.csv("Burns_F1_F3_modified.csv",header=T)

>row.names(OTUmat)=OTUmat[,1]

>OTUmat=OTUmat[,-1]

>taxmat=read.csv("OTU_map.csv",header=T)

>taxmat=as.matrix(taxmat)

>rownames(taxmat)=rownames(OTUmat)

>OTU=otu_table(OTUmat,taxa_are_rows=TRUE)

>tax=tax_table(taxmat)

>Site=c(rep("Burns",18))

>Year=c(rep("F1",6),rep("F2",6),rep("F3",6))

>Tissue=c(rep("SG",3),rep("MG",3),rep("SG",3),rep("MG",3),rep("SG",3),rep("MG",3))

>mysample_mat=matrix(cbind(Site,Year,Tissue),nrow=ncol(OTUmat),ncol=3)

>rownames(mysample_mat)=colnames(OTUmat)

>colnames(mysample_mat)=c("Site","Year","Tissue")

>mysample_mat=as.data.frame(mysample_mat)

>mysample=sample_data(as.data.frame(mysample_mat))

>physeq=phyloseq(OTU,tax,mysample)

>plot_bar(physeq,fill="Species")

>physeq2=transform_sample_counts(physeq,function(x) x/sum(x))

>plot_bar(physeq2,"Tissue",fill="Species") + facet_wrap(~Site*Year)

>plot_heatmap(physeq)

>plot_heatmap(physeq,sample.order="Site")

>plot_richness(physeq)

>my_ord=ordinate(physeq,"NMDS")

>p1=plot_ordination(physeq,my_ord,shape="Year",color="Tissue") + geom_point(size=8) + scale_colour_manual(values=c("black","red"))

>print(p1)

**ADONIS on Burns F1-F3**

>my_adonis=adonis(t(OTUmat) ~ Year*Tissue,data=mysample_mat)

>my_adonis

**Burns F1-F3 Within MG**

>my_adonis=adonis(t(OTUmat[,c(4:6,10:12,16:18)]) ~ Year,data=mysample_mat[c(4:6,10:12,16:18),])

>my_adonis

**Burns F1-F3 Within SG**

>my_adonis=adonis(t(OTUmat[,c(1:3,7:9,13:15)]) ~ Year,data=mysample_mat[c(1:3,7:9,13:15),])

>my_adonis

**2012 F1-F3**

>OTUmat=read.csv("2012_F1_F3_modified.csv",header=T)

>row.names(OTUmat)=OTUmat[,1]

>OTUmat=OTUmat[,-1]

>taxmat=matrix(row.names(OTUmat),nrow=nrow(OTUmat),ncol=1)

>rownames(taxmat)=rownames(OTUmat)

>colnames(taxmat)="Species"

>OTU=otu_table(OTUmat,taxa_are_rows=TRUE)

>tax=tax_table(taxmat)

>Site=c(rep("Burns",18), rep("Lake Como", 18))

>Year=c(rep("F1",6),rep("F2",6),rep("F3",6),rep("F1",6),rep("F2",6),rep("F3",6))

>Tissue=c(rep("SG",3),rep("MG",3),rep("SG",3),rep("MG",3),rep("SG",3),rep("MG",3),rep("SG",3),rep("MG",3),rep("SG",3),rep("MG",3),rep("SG",3),rep("MG",3))

>mysample_mat=matrix(cbind(Site,Year,Tissue),nrow=ncol(OTUmat),ncol=3)

>rownames(mysample_mat)=colnames(OTUmat)

>colnames(mysample_mat)=c("Site","Year","Tissue")

>mysample_mat=as.data.frame(mysample_mat)

>mysample=sample_data(as.data.frame(mysample_mat))

>physeq=phyloseq(OTU,tax,mysample)

>plot_bar(physeq,fill="Species")

>plot_heatmap(physeq)

>plot_richness(physeq)

**Lake Como FO vs. F1**

>OTUmat=read.csv("LC_F0_F1.csv",header=T)

>row.names(OTUmat)=OTUmat[,1]

>OTUmat=OTUmat[,-1]

>taxmat=matrix(row.names(OTUmat),nrow=nrow(OTUmat),ncol=1)

>rownames(taxmat)=rownames(OTUmat)

>colnames(taxmat)="Species"

>OTU=otu_table(OTUmat,taxa_are_rows=TRUE)

>tax=tax_table(taxmat)

>Site=c(rep("Lake Como", 22))

>Year=c(rep("F0",10),rep("F1",12))

>Tissue=c(rep("SG",5),rep("MG",5),rep("SG",6),rep("MG",6))

>mysample_mat=matrix(cbind(Site,Year,Tissue),nrow=ncol(OTUmat),ncol=3)

>rownames(mysample_mat)=colnames(OTUmat)

>colnames(mysample_mat)=c("Site","Year","Tissue")

>mysample_mat=as.data.frame(mysample_mat)

>mysample=sample_data(as.data.frame(mysample_mat))

>physeq=phyloseq(OTU,tax,mysample)

>plot_bar(physeq,fill="Species")

>plot_heatmap(physeq)

**Burns FO vs. F1**

>OTUmat=read.csv("B_F0_F1.csv",header=T)

>row.names(OTUmat)=OTUmat[,1]

>OTUmat=OTUmat[,-1]

>taxmat=matrix(row.names(OTUmat),nrow=nrow(OTUmat),ncol=1)

>rownames(taxmat)=rownames(OTUmat)

>colnames(taxmat)="Species"

>OTU=otu_table(OTUmat,taxa_are_rows=TRUE)

>tax=tax_table(taxmat)

>Site=c(rep("Burns", 24))

>Year=c(rep("F0",12),rep("F1",12))

>Tissue=c(rep("SG",6),rep("MG",6),rep("SG",6),rep("MG",6))

>mysample_mat=matrix(cbind(Site,Year,Tissue),nrow=ncol(OTUmat),ncol=3)

>rownames(mysample_mat)=colnames(OTUmat)

>colnames(mysample_mat)=c("Site","Year","Tissue")

>mysample_mat=as.data.frame(mysample_mat)

>mysample=sample_data(as.data.frame(mysample_mat))

>physeq=phyloseq(OTU,tax,mysample)

>plot_bar(physeq,fill="Species")

>plot_heatmap(physeq)

>points(myNMDS,display="species",col=c(rep("blue",5),rep("green",5),rep("red",6),rep("black",6)))

legend("bottomright",legend=c("LC F0 SG","LC F0 MG","LC F1 SG","LC F1 MG"), col=c("blue","green","red","black"),pch=rep(1:1,6),bty="n",cex=0.6)

**Rarefaction curves**

>library(vegan)

>sequences<-read.csv(‘file name’, row.names=1)

>source("C:\\Program Files\\R\\functions\\rarefaction.txt")

> rarefaction(x, subsample=5, plot=TRUE, color=TRUE, error=FALSE, legend=TRUE, symbol)
