# Supplementary material for: Laboratory colonization stabilizes the naturally dynamic microbiome composition of field collected Dermacentor andersoni ticks
Source: Microbiome. 2017 Oct 4;5:133. doi: 10.1186/s40168-017-0352-9 (PMC5628422; doi:10.1186/s40168-017-0352-9)
Supplement: Supplementary file 3 — Representative rarefaction curves of D. andersoni ticks in 2012–2014 Lake Como populations. The species diversity of the midgut (MG) and salivary glands (SG) of adult male F1 Lake Como (LC) ticks collected from 2012 to 2014 were plotted as a function of read depth. Samples that were plotted include: 2014 SG (yellow), 2014 MG (dark blue), 2012 MG (red), 2012 SG (pale blue), 2013 SG (fuchsia) and 2013 MG (green). (DOCX 53 kb) [file 40168_2017_352_MOESM3_ESM.docx]

**
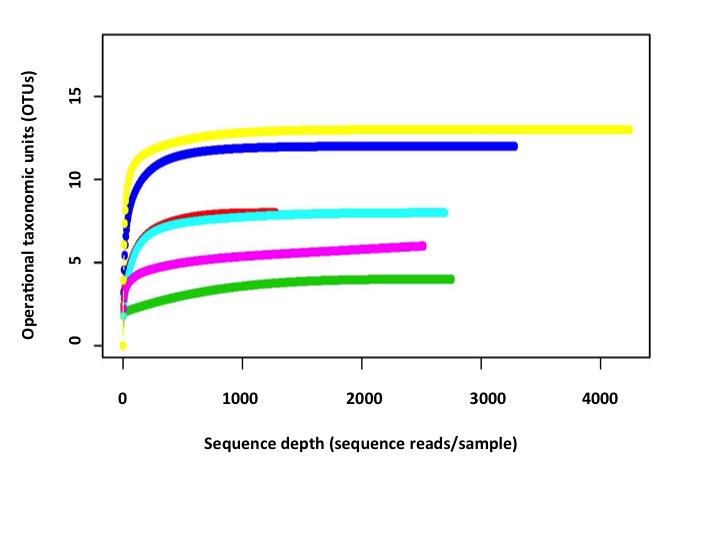
**

**Additional File 3: Representative rarefaction curves of *D. andersoni* ticks in 2012 – 2014 Lake Como populations.** The species diversity of the midgut (MG) and salivary glands (SG) of adult male F1 Lake Como (LC) ticks collected from 2012 – 2014 were plotted as a function of read depth. Samples that were plotted include: 2014 SG (yellow), 2014 MG (dark blue), 2012 MG (red), 2012 SG (pale blue), 2013 SG (fuchsia) and 2013 MG (green).
